# HexR Transcription Factor Contributes to *Pseudomonas cannabina* pv. *alisalensis* Virulence by Coordinating Type Three Secretion System Genes

**DOI:** 10.3390/microorganisms11041025

**Published:** 2023-04-14

**Authors:** Nanami Sakata, Takashi Fujikawa, Ayaka Uke, Takako Ishiga, Yuki Ichinose, Yasuhiro Ishiga

**Affiliations:** 1 Faculty of Life and Environmental Sciences, University of Tsukuba, 1-1-1 Tennodai, Tsukuba 305-8572, Ibaraki, Japan; 2 Institute of Plant Protection, National Agriculture and Food Research Organization (NARO), Tsukuba 305-8666, Ibaraki, Japan; ftakashi@affrc.go.jp; 3 Biological Resources and Post-Harvest Division, Japan International Research Center for Agricultural Sciences (JIRCAS), Tsukuba 305-8686, Ibaraki, Japan; uke@affrc.go.jp; 4 Tsukuba-Plant Innovation Research Center (T-PIRC), University of Tsukuba, Tsukuba 305-8572, Ibaraki, Japan; ishiga.takako.kb@un.tsukuba.ac.jp; 5 Graduate School of Environmental, Life, Natural Science and Technology, Okayama University, Okayama 700-8530, Okayama, Japan; yuki@okayama-u.ac.jp

**Keywords:** bacterial plant pathogen, *Pseudomonas cannabina* pv. *alisalensis*, cabbage, HexR, type three secretion system, plant immunity

## Abstract

*Pseudomonas cannabina* pv. *alisalensis* (*Pcal*) causes bacterial blight on cabbage. We previously conducted a screening for reduced virulence using Tn*5* transposon mutants and identified one of the transcriptional factors, HexR, as a potential *Pcal* virulence factor. However, the role of HexR in plant pathogenic *Pseudomonas* virulence has not been investigated well. Here, we demonstrated that the *Pcal hexR* mutant showed reduced disease symptoms and bacterial populations on cabbage, indicating that HexR contributes to *Pcal* virulence. We used RNA-seq analysis to characterize the genes regulated by HexR. We found that several type three secretion system (T3SS)-related genes had lower expression of the *Pcal hexR* mutant. Five genes were related to T3SS machinery, two genes were related to type three helper proteins, and three genes encoded type three effectors (T3Es). We also confirmed that T3SS-related genes, including *hrpL*, *avrPto*, *hopM1*, and *avrE1*, were also down-regulated in the *Pcal hexR* mutant both in culture and in vivo by using RT-qPCR. T3SS functions to suppress plant defense in host plants and induce hypersensitive response (HR) cell death in non-host plants. Therefore, we investigated the expression profiles of cabbage defense-related genes, including *PR1* and *PR5*, and found that the expressions of these genes were greater in the *Pcal hexR* mutant. We also demonstrated that the *hexR* mutant did not induce HR cell death in non-host plants, indicating that HexR contributes in causing HR in nonhost plants. Together, these results indicate that the mutation in *hexR* leads to a reduction in the T3SS-related gene expression and thus an impairment in plant defense suppression, reducing *Pcal* virulence.

## 1. Introduction

In natural habitats, plants are constantly surrounded by an enormous number of microorganisms, including potential pathogens. Therefore, plants have developed monitoring systems that recognize invading pathogens and regulate immune responses to defend themselves against pathogens. The first line of defense is pathogen-associated molecular patterns (PAMPs)-triggered immunity (PTI). PTI is initiated after recognition of PAMPs, including flagellin and elongation factor Tu (EF-Tu), using plasma membrane-localized pattern recognition receptors (PRRs) such as FLAGELLIN-SENSING2 (FLS2) and EF-Tu RECEPTOR (EFR), respectively [1,2]. While most non-adapted pathogens cannot overcome PTI, adapted pathogens secrete effectors into the plant cell that facilitate pathogen infection by, for instance, interfering with PTI [3]. The second line of defense is effector-triggered immunity (ETI). ETI is triggered by specific recognition of effectors by resistance (R) proteins [4].

*Pseudomonas syringae* are a Gram-negative γ-proteobacteria that infect and cause diseases in plants. So far, approximately 60 pathovars have been identified in the plant pathogenic *Pseudomonas* species that cause various disease symptoms, including blight, cankers, leaf spots, and galls, on different plant species [5]. In a successful disease cycle, *Pseudomonas* species generally live two lifestyles: an initial epiphytic phase on the healthy leaf surfaces, and an endophytic phase in the apoplastic space after entering the plant through natural opening sites [6,7]. One of the major virulence factors in plant pathogenic *Pseudomonas* species is type three effectors (T3Es) that are delivered into the host through a type three secretion system (T3SS) to suppress host defense responses and facilitate disease development. Plant pathogenic *Pseudomonas* species have from 9 to 39 potential T3Es [8]. Some T3Es directly interact with and disrupt the function of plant cell surface receptors and/or co-receptors that are involved in the recognition of PAMPs [9,10]. Other T3Es promote pathogen entry into the plant interior space by reopening stomata, through interaction with plant immune regulators such as RPM1-INTERACTING PROTEIN4 (RIN4) or the modulation of the plant hormone jasmonic acid [11,12,13]. Moreover, T3Es promote pathogen apoplastic fitness by stimulating water release from host cells [14]. During successful infection, bacterial pathogens need to regulate these virulence factors to adapt to various environmental changes. The extracytoplasmic function (ECF) sigma factors are one of the systems providing this induction and are capable of inducing gene sets in response to environmental stimuli [15]. One of the ECF sigma factors HrpL (hypersensitive response and pathogenicity) is a master regulator of the T3SS. The *hrpL* transcription is directed by the σ^54^ sigma factor (RpoN) and the enhancer-binding proteins HrpR and HrpS. Moreover, the *P. syringae* pv. *tomato* (*Pst*) DC3000 *hrpL* mutant is defective in coronatine (COR) production, which is one of the phytotoxins [16]. COR consists of two distinct structural components: the polyketide coronafacic acid (CFA), and coronamic acid (CMA) [17,18]. The *corR* expression, which is a response regulator required for gene expression related to CFA and CMA synthesis, was significantly less in the *hrpL* mutant, indicating that HrpL regulates COR production by modulating *corR* expression [16]. Furthermore, CorR specifically binds to the *hrpL* upstream region, indicating that CorR also regulates *hrpL* transcription [16].

*P. cannabina* pv. *alisalensis* (*Pcal*) causes bacterial blight on Brassicaceae [19]. Severe outbreaks of leaf spot and blight symptoms have been observed on cabbage, pak choi, broccoli, Chinese cabbage, red cabbage, and green ball cabbage in Japan since 2009 [19]. To identify *Pcal* virulence mechanisms, we previously conducted a screening with reduced virulence using Tn*5* transposon mutants [20], and identified several potential virulence factors, including T3SS, membrane transporters, an enzyme for amino acid metabolism, and transcriptional factors [20,21,22]. Among these mutants, a *hexR* mutant showed reduced virulence [20]. HexR is one of the transcriptional regulators and regulates glucose metabolism in *P. aeruginosa* and *P. putida* [23,24]. In plant pathogenic *Pseudomonas*, Mehmood et al. (2015) [25] demonstrated that the bacterial populations were not significantly different between wild-type *Pseudomonas savastanoi* pv. *glycinea* (*Psg*) and the *hexR* mutant, suggesting that HexR does not contribute to *Psg* virulence. Thus, we hypothesized that HexR in *Pcal* functions differentially from *Psg* and contributes to its virulence. Therefore, we at this point decided to investigate the HexR roles in plant pathogenic bacterial virulence, especially in *Pcal* virulence.

In this study, we first conducted RNA-seq analysis and identified that HexR coordinates the expression of T3SS-related genes, which is one of the major *Pcal* virulence factors. We confirmed that T3SS-related genes were regulated in culture and during infection by using RT-qPCR. Moreover, we also demonstrated that the expressions of cabbage defense- related genes were greater in the *Pcal hexR* mutant compared to WT. Together, our results suggest that the down-regulation of T3SS-related genes in the *Pcal hexR* mutant leads to the impairment of plant defense suppression, reducing *Pcal* virulence.

## 2. Materials and Methods

### 2.1. Bacterial Strains, Plasmids, and Growth Conditions

The bacterial strains and plasmids used in this study are described in Table S1. *Pseudomonas cannabina* pv. *alisalensis* strain KB211 (*Pcal* KB211) was used as the pathogenic strain. *Pcal* wild-type (WT) was grown on King’s B (KB; [26]) medium at 28 °C. The *hexR* mutant was grown on KB containing kanamycin (Km) (10 µg/mL). The *hexR* mutant complemented with pDSKG-*hexR* was grown on KB containing Km (10 µg/mL) and gentamicin (Gen) (25 µg/mL) (Table S1). *Pseudomonas syringae* pv. *tomato* DC3000 (*Pst* DC3000) was used as the pathogenic strain on *Arabidopsis thaliana*. *Pst* wild-type (WT) and Δ*hexR* was grown on KB medium at 28 °C. Before *Pcal* and *Pst* inoculation, bacteria were suspended in sterile distilled H_2_O, and the bacterial cell densities at 600 nm (OD_600_) were measured using a Biowave CO8000 Cell Density Meter (Funakoshi, Tokyo, Japan).

### 2.2. Complementation of the hexR Mutant

The *hexR*-complemented strain was constructed as described in Ishiga et al. (2018) [27]. Briefly, the pDSKG vector [28] replaced the kanamycin cassette to gentamycin. The *hexR* was transferred into the pDSKG vector to generate pDSKG-*hexR*. The pDSKG-*hexR* construct was introduced into the *hexR* mutant by electroporation to generate the complemented strain.

### 2.3. Generation of the Pseudomonas syringae pv. tomato ΔhexR Mutant

The mutant was generated as described previously (Ishiga et al. 2018) [27]. Briefly, the genetic regions containing *hexR* and surrounding regions were amplified using PCR primer sets (Table S2) and inserted into the pGEM-T Easy vector (Promega, Madison, WI, USA). The inverse PCR was carried out, using a primer set (Table S2), to delete the *hexR* open reading frame. After digesting with BamHI and DpnI, the resultant DNA was self-ligated with T4 DNA ligase (Ligation-Convenience kit, Nippon Gene, Tokyo). The *hexR*-deleted DNA constructs were introduced into pK18*mobsacB* [29] and then transformed into *E. coli* S17-1. The deletion mutant was obtained by conjugation and homologous recombination according to the method previously reported [30].

### 2.4. Bacterial In Vitro Growth Measurements

*Pcal* WT, the *Pcal hexR* mutant, and the *Pcal hexR* mutant complemented with pDSKG-*hexR* were grown at 28 °C on KB medium. The bacterial suspensions were standardized to an OD_600_ of 0.01 with KB, and bacterial growth was measured at OD_600_ for 24 h.

### 2.5. Plant Materials

Cabbage (*Brassica oleracea* var. *capitata*) cv. Kinkei 201 was used for *Pcal* virulence assays. All plants were grown from seed at 23–25 °C with a light intensity of 200 μEm^−2^s^−1^ and a 16 h light/8 h dark photoperiod.

*A. thaliana* (Col-0) was used for *Pst* virulence assays. *A. thaliana* seeds were germinated and grown on 1/2 strength Murashige and Skoog (MS) medium (0.3% phytagel) with Gamborg vitamins (Sigma-Aldrich, St. Louis, MO, USA). Seedlings were incubated in a growth chamber at 24 °C with a light intensity of 200 μEm^−2^s^−1^ and a 12 h light/12 h dark photoperiod.

*Nicotiana tabacum* var. Xanthi was used for hypersensitive reaction (HR) cell assays. The plants were grown from seed at 23–25 °C with a light intensity of 200 μEm^−2^s^−1^ and a 16 h light/8 h dark photoperiod.

### 2.6. Bacterial Inoculations on Cabbage and A. thaliana

To assay for cabbage disease, dip inoculation was conducted by soaking seedlings with bacterial suspensions (5 × 10^7^ colony-forming units: CFU/mL) containing 0.025% Silwet L-77 (OSI Specialities, Danbury, CT, USA). For syringe inoculation, plants were inoculated with bacterial suspensions (5 × 10^4^ CFU/mL) with a 1 mL blunt syringe into leaves. Flood inoculation was conducted as described previously [31]. Briefly, 50 mL of bacterial suspension (1 × 10^8^ CFU/mL) made in sterile distilled H_2_O containing 0.025% Silwet L-77 (OSI Specialities) was dispensed onto a plate containing 2-week-old *A. thaliana* seedlings grown on Murashige and Skoog (MS) medium for uniform inoculation.

### 2.7. Bacterial Populations Measurements

To assess bacterial growth in plants, the internal bacterial populations in the plant were evaluated. Dip- and flood-inoculated leaves were surface-sterilized with 10% H_2_O_2_ for 3 min. After washing with sterile distilled water, the leaves were homogenized in sterile distilled water, and diluted samples were plated onto solid KB agar medium. The bacterial populations at 0 dpi were estimated using leaves harvested 1 h post-inoculation (hpi) without surface sterilization. For syringe inoculation, to assess bacterial growth in cabbage, leaf discs were harvested using a cork borer. The plant samples were homogenized in sterile distilled water, and diluted samples were plated onto solid KB agar medium. The bacterial colony-forming units (CFUs) were counted and normalized as CFU per gram or CFU per cm^2^, using the total leaf weight or leaf square centimeters. The bacterial populations were evaluated in at least three independent experiments.

### 2.8. RNA Purification

For expression profiles of *Pcal* WT and *hexR* mutant genes in culture, bacteria were grown in KB broth for 24 h, then adjusted to an OD_600_ of 0.3 with fresh KB broth and grown for 3 h further, and then incubated in MG medium for 30 min. Total RNA was extracted using Reliaprep (Promega) according to the manufacturer’s protocol.

To analyze *Pcal* and cabbage gene expression profiles during infection, we dip-inoculated cabbage plants with *Pcal* at 1 × 10^8^ CFU/mL, and at 4 and 48 h the total RNAs including plant and bacterial RNAs were extracted from infected leaves and purified using RNAiso Plus (Takara Bio, Kusatsu, Japan).

### 2.9. RNA-Seq Analysis

After total RNAs were isolated from *Pcal* WT and the *hexR* mutant, rRNAs were almost depleted from the total RNAs by using the RiboMinus Transcriptome Isolation Kit for bacteria (Thermo Fisher Scientific Inc., Waltham, MA, USA). The RNA-Seq library was constructed from sample RNAs with the Ion Total RNA-Seq Kit v2 (Thermo Fisher Scientific Inc., Waltham, MA, USA) and Ion Xpress RNA-Seq barcode (Thermo Fisher Scientific Inc., Waltham, MA, USA). Subsequently, using the Ion 540 Chef Kit on an Ion Chef system (Thermo Fisher Scientific Inc., Waltham, MA, USA), RNA-Seq templates were prepared. Sequencing of the amplicon libraries was performed using an Ion 540 Chip with the Ion GeneStudio S5 system (Thermo Fisher Scientific Inc., Waltham, MA, USA). Sequence data were assembled and analyzed with the CLC Genomics Workbench (Qiagen, Valencia, CA, USA). The *P. syringae* pv. *maculicola* ES4326 genome sequence (GeneBank accession number: CP047260) was used as the reference for RNA-Seq mapping and assembly of sequence reads. The gene expression value was calculated from the ratio of the number of mapped reads to “reads per kilobase million (RPKM)” in *Pcal* WT and the *hexR* mutant. Since the mutant strain used in this study might affect the expression profiles of the downstream genes, the analysis was conducted by using RPKM, which is more suitable for characterizing each strain compared to “transcripts per million (TPM)”. The subsequent mathematical analyses were all performed by the R program as follows: expression ratio of the *hexR* mutant against *Pcal* WT was calculated using the GLM method implemented in the edgeR package according to McCarthy et al. (2021) [32]. At this time statistical data were obtained by one-way ANOVA. The adjusted *p*-value and FDR values shown in this study are cited values of these statistical results. Additionally, as is well known, when the sample parameter is small or the number of iterations is small, the *p*-value tends to be large [33]. Moreover, because expression comparison is performed by a large number of genes between *Pcal* WT and the *hexR* mutant in this study, the *p*-value may become large. Thus, the second type of error in statistical analysis (Type II error), which produces false negative results, is likely to occur. Therefore, to avoid a Type II error as much as possible, genes were filtered at 0.1 of permissive FDR value, and the effect size (eta-squared) was calculated and attached to the statistical data of each gene expression. The eta-squared was calculated by the R program using the etaSquared method implemented in the Isr package.

### 2.10. RT-qPCR

The DNase-treated RNA was reverse-transcribed using the ReverTra Ace qPCR RT Master Mix (TOYOBO, Osaka, Japan). The cDNA (1:10) was then used for RT-qPCR using the primers shown in Table S2 with THUNDERBIRD SYBR qPCR Mix (TOYOBO) on a Thermal Cycler Dice Real Time System (Takara Bio). *Pcal* KB211 *outer membrane porin F* (*oprF*) and *recombinase A* (*recA*) were used to normalize gene expression. Cabbage *UBIQUITIN EXTENSION PROTEIN 1* (*BoUBQ1*) was used as an internal control to normalize gene expression.

### 2.11. Hypertrophy-Inducing Activity Assay on Potato Tuber Tissue

Hypertrophy-inducing activity assay was conducted as described in Nguyen et al. (2021) [28]. Briefly, potato tuber discs were inoculated using toothpicks by placing the tip in *Pcal* WT and the *hexR* mutant on a KB medium plate, and then placing the toothpick on the potato tuber disc. The discs were then placed at 23 °C in an incubator (darkness) for 5 days. Photographs were taken at 5 dpi.

### 2.12. Hypersensitive Response Assays

For HR assays, 2-month-old *Nicotiana tabacum* var. Xanthi plants were used. The leaves were syringe-infiltrated with *Pcal* WT and the *hexR* mutant with an OD_600_ of 0.1 (5 × 10^7^ CFU/mL). HR cell death symptoms were photographed at 24 hpi.

## 3. Results

### 3.1. Importance of HexR in Pseudomonas Virulence

**Figure 1:**
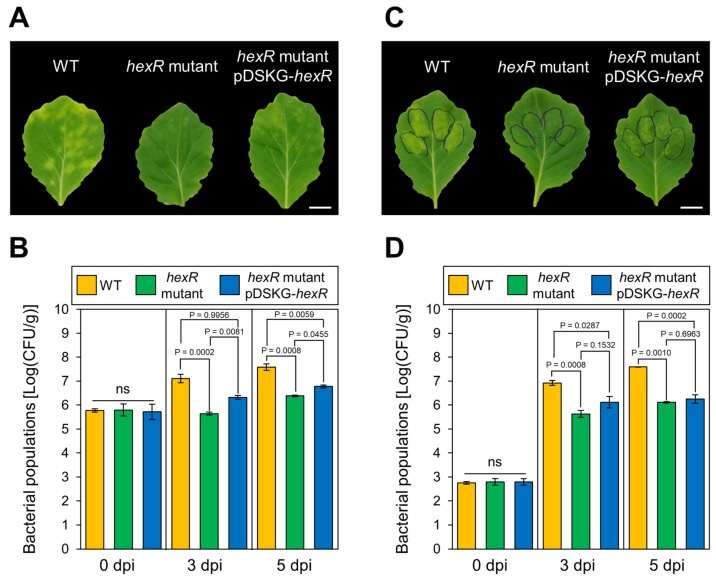
Disease phenotypes and bacterial populations of *Pseudomonas cannabina* pv. *alisalensis* KB211 WT and the *hexR* mutant in cabbage after dip and syringe inoculation. Disease symptoms (**A**) and bacterial populations (**B**) in cabbage dip-inoculated with WT, the *hexR* mutant, and the *hexR* mutant complemented with pDSKG-*hexR*. Disease symptoms (**C**) and bacterial populations (**D**) in cabbage syringe-inoculated with WT, the *hexR* mutant, and the *hexR* mutant complemented with pDSKG-*hexR*. Cabbage was dip-inoculated with 5 × 10^7^ CFU/mL of inoculum containing 0.025% SilwetL-77 and syringe-inoculated with 5 × 10^4^ CFU/mL of inoculum, respectively. Bacterial populations in the plant leaves were evaluated at 0, 3, and 5 dpi. The leaves were photographed 5 days after dip inoculation, and 3 days after syringe inoculation. Vertical bars indicate the standard error for at least three independent experiments. ns indicates not significant. Statistical analysis was performed using one-way ANOVA with Tukey’s HSD test. Scale bar shows 1 cm.

We first measured the *Pseudomonas cannabina* pv. *alisalensis* (*Pcal*) WT, *hexR* mutant, and *hexR*-complemented strain growth in vitro. The *Pcal hexR* mutant tended to be defective in growth in comparison with that of *Pcal* WT and the complemented strain at 24 h and 48 h after incubation in KB media (Figure S1). To investigate the importance of HexR in *Pcal* virulence, we challenged cabbage with *Pcal* WT, the *hexR* mutant, and the *hexR*-complemented strain by dip inoculation. Cabbage leaves inoculated with *Pcal* WT showed severe chlorosis, but the *Pcal hexR* mutant showed no symptoms (Figure 1A), indicating that the *Pcal hexR* mutant had lost its pathogenicity. Moreover, bacterial populations of the *Pcal hexR* mutant were significantly reduced compared to *Pcal* WT and the complemented strain (Figure 1B). To further investigate whether HexR is important for virulence in the intercellular space (apoplast), we also conducted syringe inoculation. When infiltrated directly into the cabbage apoplast, the *Pcal hexR* mutant also showed no symptoms and reduced bacterial populations compared to *Pcal* WT and the complemented strain (Figure 1C,D). The disease symptoms and bacterial populations of the complemented strains were greater than that of the *Pcal hexR* mutant, but less than that of *Pcal* WT, indicating that the complemented strains partially restored their virulence (Figure 1A–D). Together, these results indicate that HexR is required for *Pcal* virulence in cabbage.

We also investigated the importance of HexR in the virulence of another plant pathogenic *Pseudomonas*. Therefore, we conducted inoculation assays with *Pseudononas syringae* pv. *tomato* (*Pst*) DC3000 WT and the *hexR* mutant (Δ*hexR*) on *A. thaliana*. Disease symptoms caused by *Pst* WT showed severe chlorosis (Figure S2A). Although disease symptoms by *Pst* Δ*hexR* showed less chlorosis compared to *Pst* WT, the *Pst* Δ*hexR* also still caused disease symptoms (Figure S2A). Bacterial populations did not show any significant differences between *Pst* WT and Δ*hexR* (Figure S2B). These results suggest that HexR does not contribute to *Pst* virulence in *A. thaliana*.

### 3.2. Gene Expression Profiles of Pseudomonas cannabina pv. alisalensis WT and the hexR Mutant

To compare gene expression profiles, *Pcal* WT and the *hexR* mutant were incubated in culture, and the complete transcriptome was determined for each strain using RNA-seq analysis. This analysis identified 21 HexR-dependent genes for which expression changed by twofold or more and had FDR values of less than 1% (Table 1). Several genes that encode genes related to the type three secretion system (T3SS), coronatine (COR), ABC transporter, and auxin biosynthesis, had less expression in the *Pcal hexR* mutant compared to *Pcal* WT (Table 1). The expression of several T3SS-related genes was suppressed in the *Pcal hexR* mutant compared to *Pcal* WT (Table 1). Five genes were related to T3SS machinery, two genes were related to type three helper proteins, and three genes encoded type three effectors (T3Es) (Table 1). Moreover, *hrpL*, which encodes an alternate RNA polymerase sigma factor required for the expression of T3SS genes, was suppressed in the *Pcal hexR* mutant (Table 1). Additionally, *corR*, which encodes a response regulator transcriptional factor required for the expression of COR biosynthesis genes, was also suppressed in the *Pcal hexR* mutant compared to *Pcal* WT (Table 1). Therefore, we focused on genes related to the T3SS and COR.

**Figure 2:**
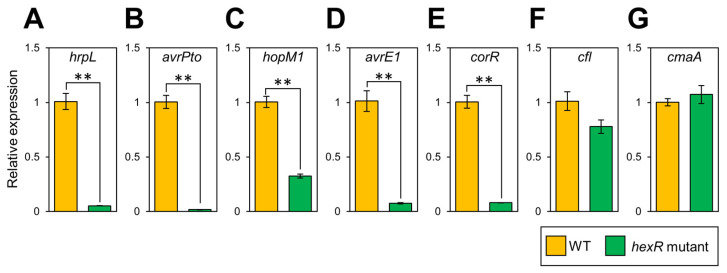
Bacterial virulence gene expression profiles in *Pseudomonas cannabina* pv. *alisalensis* KB211 WT and the *hexR* mutant in culture. Bacteria were grown in KB broth for 24 h, then adjusted to an OD_600_ of 0.3 with fresh KB broth and grown for 3 h further and then incubated in MG medium for 30 min. Expression profiles of type three secretion system-related genes (including *hrpL* (**A**), *avrPto* (**B**), *hopM1* (**C**), and *avrE1* (**D**)) and COR-biosynthesis-related genes (including *corR* (**E**), *cfl* (**F**), and *cmaA* (**G**)) were investigated. Total RNA was extracted for use in real-time quantitative reverse transcription–polymerase chain reaction (RT-qPCR) with gene-specific primer sets (Table S2). Expression was normalized using *oprE* and *recA*. Vertical bars indicate the standard error for at least six biological replicates. Asterisks indicate a significant difference from WT in a *t*-test (** *p* < 0.01).

We next confirmed the gene expression profiles in culture by using RT-qPCR. The T3SS-related genes, including *hrpL*, *avrPto*, *hopM1*, and *avrE1*, were all down-regulated in the *Pcal hexR* mutant (Figure 2A–D). *corR* gene expression was also significantly reduced, but that of *cmaA* and *cfl* was not down-regulated (Figure 2E–G). We also investigated COR production by using a hypertrophy-inducing activity test on potato tuber tissues [34]. While *Pcal* Δ*cmaA*, which is a deletion mutant for a COR biosynthesis gene, showed no hypertrophy response, the *Pcal* WT and *hexR* mutant-inoculated potato tuber tissues showed hypertrophy response (Figure S3), indicating that the *Pcal hexR* mutant was not impaired in COR production.

**Figure 3:**
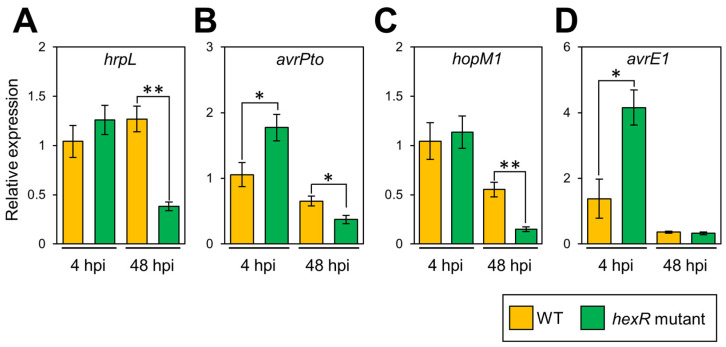
Bacterial virulence gene expression profiles in *Pseudomonas cannabina* pv. *alisalensis* KB211 WT and the *hexR* mutant in vivo. Cabbage plants were syringe inoculated with 5 × 10^7^ CFU/mL of WT and the *hexR* mutant and total RNAs were collected 4 and 48 hpi. Expression profiles of type three secretion system-related genes (including *hrpL* (**A**), *avrPto* (**B**), *hopM1* (**C**), and *avrE1* (**D**)) were investigated. Total RNA was extracted for use in real-time quantitative reverse transcription–polymerase chain reaction (RT-qPCR) with gene-specific primer sets (Table S2). Expression was normalized using *oprE* and *recA*. Vertical bars indicate the standard error for at least six biological replicates. Asterisks indicate a significant difference from WT in a *t*-test (* *p* < 0.05, ** *p* < 0.01).

Since the genes related to COR biosynthesis, *cmaA* and *cfl*, were not regulated in culture and the *Pcal hexR* mutant did not abrogate COR production, we investigated the gene expression profiles of T3SS-related genes, including *hrpL*, *avrPto*, *hopM1*, and *avrE1*, during infection. The expression of *hrpL* and *hopM1* were not significantly different between *Pcal* WT and the *hexR* mutant at 4 hpi, but were down-regulated in the *Pcal hexR* mutant at 48 hpi (Figure 3A,C). Furthermore, the expressions of *avrPto* and *avrE1* were up-regulated in the *Pcal hexR* mutant at 4 hpi (Figure 3B,D). These results indicate that HexR regulates the expression of T3SS-related genes.

### 3.3. Gene Expression Profiles of Plant Defense-Related Genes Inoculated with Pseudomonas cannabina pv. alisalensis WT and the hexR Mutant

**Figure 4:**
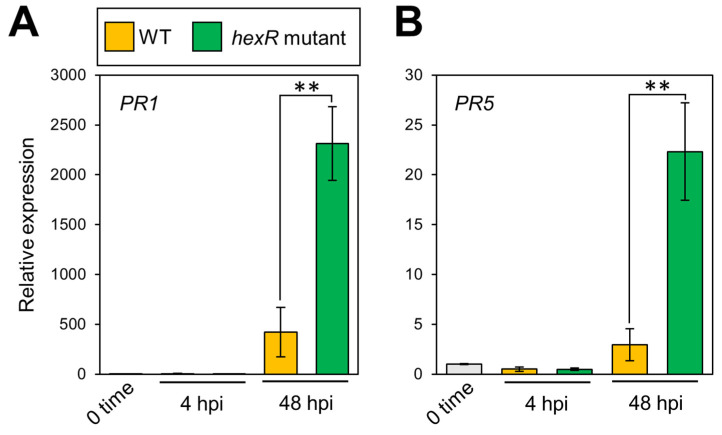
Expression profiles of cabbage defense-related genes. Cabbage plants were syringe inoculated with 5 × 10^7^ CFU/mL of WT and the *hexR* mutant, and total RNAs were collected at 4 and 48 hpi. Expression profiles of cabbage defense-related genes (including *PR1* (**A**), and *PR5* (**B**)) were investigated. Total RNA was extracted for use in real-time quantitative reverse transcription–polymerase chain reaction (RT-qPCR) with gene-specific primer sets (Table S2). Expression was normalized using *BoUBQ*. Vertical bars indicate the standard error for at least six biological replicates. Asterisks indicate a significant difference from WT in a *t*-test (** *p* < 0.01).

Since T3SS functions to suppress plant defense, we hypothesized that the down-regulation of T3SS-related genes in the *Pcal hexR* mutant would lead to impairment in plant defense suppression. Thus, we next investigated plant defense-related gene expression, including *PR1* and *PR5*. The expressions of both genes were significantly greater in the *Pcal hexR* mutant compared to *Pcal* WT (Figure 4A,B). These results indicate that HexR contributes to suppressing plant defense during infection.

### 3.4. Hypersensitive Response in Nonhost Plants Inoculated with Pseudomonas cannabina pv. alisalensis WT and the hexR Mutant

**Figure 5:**
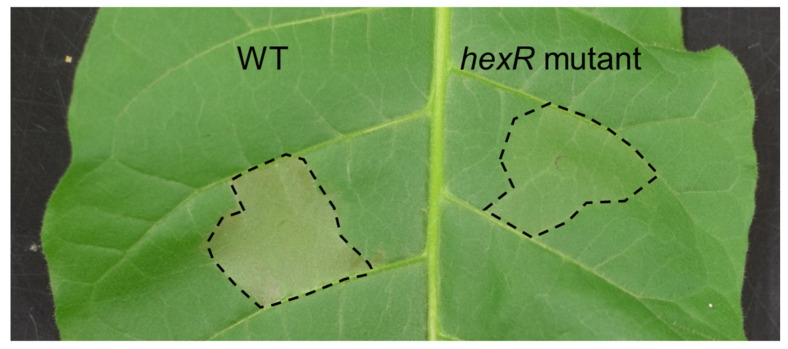
Induction of hypersensitive response cell death caused by *Pseudomonas cannabina* pv. *alisalensis* KB211 WT and the *hexR* mutant on tobacco leaves. WT and the *hexR* mutant were syringe inoculated with 5 × 10^7^ CFU/mL into tobacco leaves. Photos were taken at 24 h.

Hypersensitive response (HR) cell death is induced by *hrp*-associated proteins [35]. Therefore, to further investigate the impact of the *Pcal hexR* mutant on the *hrp* system, HR assays were performed on non-host tobacco (*Nicotiana tabacum*). HR cell death was induced in tobacco plants by *Pcal* WT (Figure 5). Conversely, the *Pcal hexR* mutant did not induce HR cell death (Figure 5).

## 4. Discussion

One of the transcriptional factors, HexR, was identified as a potential *Pseudomonas cannabina* pv. *alisalensis* (*Pcal*) virulence factor [20]. However, the role of HexR in plant pathogenic *Pseudomonas* virulence has not been investigated well. To investigate HexR roles in *Pcal* virulence, we firstly conducted inoculation assay and demonstrated that the *Pcal hexR* mutant showed reduced disease symptoms and bacterial populations on cabbage, indicating that HexR contributes to *Pcal* virulence. We also conducted RNA-seq analysis and revealed that several type three secretion system (T3SS)-related genes had less expression in the *Pcal hexR* mutant. Plant defense-related genes showed greater expression in cabbage inoculated with the *Pcal hexR* mutant compared to WT, suggesting that HexR contributes to suppressing plant defense. Together, these results suggest that the down-regulation of T3SS-related genes in the *Pcal hexR* mutant leads to impairment in plant defense suppression, resulting in reduced *Pcal* virulence.

The disease symptoms and bacterial populations on cabbage inoculated with the *Pcal hexR* mutant were significantly reduced compared to that of *Pcal* WT after dip and syringe inoculation (Figure 1). If HexR does not contribute to *Pcal* virulence after its entry into the plant, the *hexR* mutant would not show any significant differences compared to WT after syringe inoculation, which is an inoculation method for directly injecting bacterial suspensions into plants. Thus, these results indicate that HexR contributes to *Pcal* virulence even after *Pcal* entry into plants. Interestingly, the *Pseudomonas syringae* pv. *tomato* (*Pst*) Δ*hexR* caused similar disease symptoms to *Pst* WT in *A. thaliana*, and bacterial populations were not significantly different between *Pst* WT and Δ*hexR* (Figure S2). Similarly, in *P. savastanoi* pv. *glycinea* (*Psg*), the total populations and percentages of internalized bacteria were not significantly different between *Psg* WT and the *hexR* mutant [25]. Therefore, it is tempting to speculate that HexR contributes to virulence differently among plant pathogenic *Pseudomonas* species.

The *Pcal hexR* mutant complemented with pDSKG-*hexR* restored its virulence partially (Figure 1). Plasmid complementation often cannot fully restore virulence. The *Pcal* Δ*cmaA* showed fewer disease symptoms and bacterial populations compared to WT, indicating that Coronatine (COR) contributes to *Pcal* virulence [36]. However, the *Pcal* Δ*cmaA* complemented with pDSKG-*cmaA* did not restore its virulence (Sakata et al., unpublished data). Similarly, RND transporter contributes to diseases [22], and mutants complemented with pDSKG-PMA4326_12408 also did not restore virulence (Sakata et al., unpublished data). Besides *Pcal*, *P. amygdali* pv. *tabaci* (*Pta*) Δ*cheA1* showed reduced swimming motility and virulence in host tobacco plants, but its complementation did not recover its swimming motility and virulence [37]. Conversely, the *Pcal trpA* (tryptophan synthase alpha chain)-complemented strain fully restored its virulence [21]. One possibility is that the plasmid vector is effective only in the case of rescued housekeeping genes, such as *trpA*. Conversely, constitutive gene expression, which encodes the virulence-specific genes (e.g., *hexR*, *cmaA*, PMA4326_12408, and *cheA1*) might result in a disadvantage for bacterial growth. Further efforts to create complemented strains in different ways, such as by using a low copy plasmid or introducing it chromosomally, are needed. Although further quantitative analysis for spatiotemporal promoter activity is needed, these facts suggest that the regulation of virulence-related genes is important for successful infection.

Our RNA-seq analysis revealed that several T3SS-related genes had less expression in the *Pcal hexR* mutant compared to WT (Table 1). Moreover, the expression of *hrpL* and *corR* was less in the *Pcal hexR* mutant than that in WT (Table 1). We also demonstrated that *hrpL* and *corR* expression were down-regulated in the *Pcal hexR* mutant in culture (Figure 2). Moreover, type three effectors (T3Es), including *avrPto*, *hopM1*, and *avrE1*, had less expression in the *Pcal hexR* mutant (Figure 2). However, *cmaA* and *cfl* expression were not down-regulated in the *Pcal hexR* mutant (Figure 2). Additionally, both the *Pcal* WT and *hexR* mutant induced hypertrophy response in potato tubers (Figure S3), indicating that the *Pcal hexR* mutant was not impaired in COR production. These results suggest that HexR regulates T3Es, but does not regulate COR biosynthesis. HrpL regulates COR production by modulating *corR* expression [16]. Therefore, HexR might regulate *hrpL*, resulting in the down-regulation of *corR* gene expression.

Our results clearly demonstrated that T3SS gene expression was significantly downregulated in the *Pcal hexR* mutant (Figure 3) and that plant defense-related gene expression was not suppressed in the *Pcal hexR* mutant (Figure 4). T3SS mutants typically lose their ability to grow parasitically or be pathogenic in host plants [38]. Moreover, Xie et al. (2023) [39] demonstrated that a nonpathogenic isolate of *P. syringae* pv. *actinidiae* biovar 3 (*Psa*3) was defective in *hrpL* transcription and lost the ability to induce T3SS-dependent phenotypes. These studies suggest that the T3SS regulated by HrpL is essential for the determination of pathogenicity. Together, these results suggest that the down-regulation of the T3SS in the *Pcal hexR* mutant results in the impairment of plant defense suppression. Moreover, the *Pcal hexR* mutant did not induce hypersensitive response (HR) cell death in nonhost plants (Figure 5). Furthermore, the *Pcal hexR* mutant lost its pathogenicity on cabbage (Figure 1). These results indicate that the secretion of *hrp*-associated T3Es was suppressed when *hexR* was mutated in *Pcal*. Conversely, in *Psg*, HexR did not influence its ability to cause a HR in nonhost plants [25]. Additionally, the bacterial populations of the *Psg hexR* mutant were not significantly different compared to *Psg* WT [25]. These results suggest that the loss of pathogenicity in the *Pcal hexR* mutant was mainly caused by the impairment of T3SS regulation.

Kim et al. (2008) [23] demonstrated that HexR might be a dual-sensing regulator of zwf-1 (glucose-6-phosphate dehydrogenase) induction that is able to respond to both 2-keto-3 deoxy-6-phosphogluconate (KDPF) and oxidative stress in *P. putida*. Moreover, HexR might repress the expression of extra-cellular levansucrase in *Psg* [25]. These results demonstrated that HexR controls genes involved in glucose metabolites. However, any genes related to carbon metabolism and sucrose utilization were not identified from our RNA-seq analysis. Since the *Pcal hexR* mutant tended to be defective in growth compared to WT and the complemented strain (Figure S1), there is a possibility that a growth deficit might contribute to a reduction in virulence.

Our RNA-seq analysis also demonstrated that HexR regulates ABC transporter and auxin metabolism as well as the T3SS (Table 1). Yan et al. (2020) [40] demonstrated that an amino acid ABC transporter pathway (AatJ-AauS-AauR) in *Pst* directly regulates T3SS-encoding genes in response to host aspartate and glutamate signals. Furthermore, the plant hormone auxin (indole-3-acetic acid [IAA]) modulates virulence gene expression, including the T3SS and COR in *Pst* [41]. Further investigation into virulence factor regulation will help to understand how plant pathogenic *Pseudomonas* species coordinate the expression of multiple virulence factors under changing environmental conditions.

## 5. Conclusions

In summary, our results suggest that HexR contributes to *Pseudomonas cannabina* pv. *alisalensis* (*Pcal*) virulence by coordinating type three secretion system (T3SS) genes. The results presented here also suggest that the mutation in *hexR* leads to a reduction in T3SS-related gene expression, and thus impaired plant defense suppression, reducing *Pcal* virulence. To our knowledge, this is the first report to demonstrate the importance of HexR in plant pathogenic *Pseudomonas*. Our results also provide the possibility that the role of HexR in plant pathogenic *Pseudomonas* species is different. Therefore, further investigation of other extracytoplasmic function (ECF) sigma factors that regulate virulence factors during infection in *Pcal* and other *Pseudomonas* species will be needed to understand the further role of HexR in virulence and other regulatory mechanisms in plant-bacterial pathogen interactions.

## Figures and Tables

**Table 1:** The differential gene expression between *Pcal* WT and *hexR* mutant.

Category	Locus Tag	Gene	Function	Log_2_ Fold Changes	FDR Values
Type three secretion system	PMA4326_006560	*hrpL*	Sigma-70 family RNA polymerase sigma factor	−4.79	4.35 × 10^−21^
	PMA4326_006445	*hrpB*	Type III secretion system inner rod subunit	−5.85	5.56 × 10^−15^
	PMA4326_006450	*hrcJ*	Type III secretion inner membrane ring lipoprotein	−4.17	3.82 × 10^−8^
	PMA4326_006515	*hrcR*	Type III secretion system export apparatus protein	−5.40	4.07 × 10^−7^
	PMA4326_006475	*hrcC*	Type III secretion system outer membrane ring subunit	−3.03	0.003471
	PMA4326_006535	*hrpO*	Type III secretion protein	−4.00	0.001669
	PMA4326_006440	*hrpZ1*	Type III helper protein	−6.05	2.05 × 10^−50^
	PMA4326_006565	*hrpK*	Type III helper protein	−5.12	4.04 × 10^−7^
	PMA4326_019340	*avrPto*	Type III effector protein	−4.83	1.78 × 10^−14^
	PMA4326_003310	*hopAB2*	Type III effector protein	−2.70	1.17 × 10^−13^
	PMA4326_006570	*hopX1*	Type III effector protein	−2.78	0.006773
ABC transporter	PMA4326_020240	*aatJ*	Glutamate/aspartate ABC transporter substrate-binding protein	−2.39	7.53 × 10^−5^
	PMA4326_020255	*aatP*	Amino acid ABC transporter ATP-binding protein	−2.43	0.001669
Others	PMA4326_024665	*corR*	Response regulator transcription factor	−3.68	6.24 × 10^−6^
	PMA4326_025720	*iaaL*	AMP-binding protein	−3.50	1.06 × 10^−5^
	PMA4326_003600		Amidinotransferase	−4.83	8.93 × 10^−5^
	PMA4326_023615		DnaJ domain-containing protein	−2.79	8.93 × 10^−5^
	PMA4326_027030		DUF1127 domain-containing protein	2.20	0.000114
	PMA4326_002410		Restriction endonuclease	−5.86	2.94 × 10^−18^
Hypothetical	PMA4326_015740		Hypothetical protein	−3.75	0.006992
	PMA4326_006435		Hypothetical protein	−5.12	1.70 × 10^−24^

## Data Availability

The data that support the findings of this study are available from the corresponding author upon reasonable request.

## Supplementary Materials

The following supporting information can be downloaded at: https://www.mdpi.com/article/10.3390/microorganisms11041025/s1, Figure S1: Bacterial populations in vitro, Figure S2: Disease phenotypes and bacterial populations of *Pseudomonas syringae* pv. *tomato* DC3000 WT and the Δ*hexR* mutant in *Arabidopsis thaliana* after flood inoculation, Figure S3: Coronatine detection of *Pseudomonas cannabina* pv. *alisalnesis* KB211 WT, *hexR* mutant, and Δ*cmaA*, Table S1: Bacterial strains and plasmids used in this study, Table S2: Primer sets used in this study.
