# Supplementary material for: HexR Transcription Factor Contributes to Pseudomonas cannabina pv. alisalensis Virulence by Coordinating Type Three Secretion System Genes
Source: Microorganisms. 2023 Apr 14;11(4):1025. doi: 10.3390/microorganisms11041025 (PMC10145369; doi:10.3390/microorganisms11041025)
Supplement: Supplementary file 1 [file microorganisms-11-01025-s001.zip › Supplementary Figure.pdf]

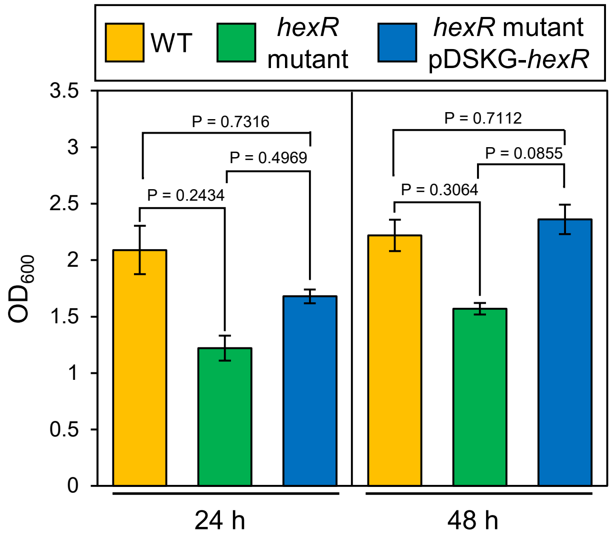

Supplementary Figure S1. Bacterial populations *in vitro*. WT and the *hexR* mutant were grown at 28 °C on KB medium. The bacterial suspensions were standardized to an OD_{600} of 0.01 with KB broth, and bacterial growth was measured at OD_{600} for 24 and 48 h. Vertical bars indicate the standard error for three biological replicates. Statistical analysis was performed using one-way ANOVA with Tukey's HSD test.

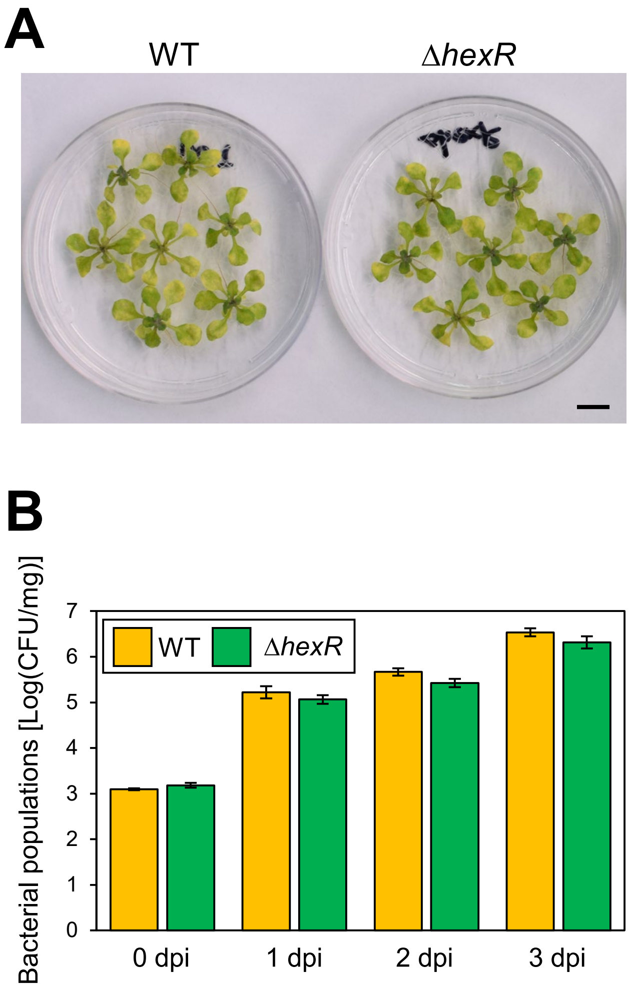

Supplementary Figure S2. Disease phenotypes and bacterial populations of *Pseudomonas syringae* pv. *tomato* DC3000 WT and the Δ*hexR* mutant in *Arabidopsis thaliana* after flood-inoculation. Disease symptoms (A) and bacterial populations (B) in *A. thaliana* flood-inoculated with WT and the Δ*hexR* mutant. *A. thaliana* was flood-inoculated with 1 x 10^8 CFU/ml of inoculum containing 0.025% Silwet L-77. Bacterial concentrations in the plant leaves were evaluated at 0, 1, 2, and 3 dpi. The leaves were photographed 3 dpi. Vertical bars indicate the standard error for at least three independent experiments. Different letters indicate a significant difference among treatments based on a Tukey's HSD test ($p < 0.05$). Scale bar shows 1 cm.

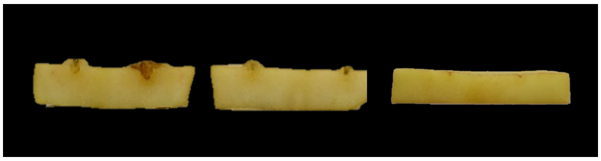

Supplementary Figure S3. Coronatine quantification of *Pseudomonas cannabina* pv. *alisalnesis* KB211 WT, *hexR* mutant, and Δ*cmaA*. Observation of hypertrophy-inducting activity on potato tuber tissue inoculated with *Pseudomonas cannabina* pv. *alisalensis* WT, *hexR* mutant, and coronatine defective mutants (Δ*cmaA*). Potato tuber discs were inoculated using toothpicks by placing the tips in the WT, *hexR* mutant, and Δ*cmaA* on a KB medium plate and then placing the toothpick on the potato tuber disc. Photographs were taken at 5 dpi.
