# Supplementary material for: HexR Transcription Factor Contributes to Pseudomonas cannabina pv. alisalensis Virulence by Coordinating Type Three Secretion System Genes
Source: Microorganisms. 2023 Apr 14;11(4):1025. doi: 10.3390/microorganisms11041025 (PMC10145369; doi:10.3390/microorganisms11041025)
Supplement: Supplementary file 1 [file microorganisms-11-01025-s001.zip › Supplementary Table.pdf]

Table S1. Bacterial strains and plasmids used in this study

Bacterial strain or plasmid	Relevant characteristics	Reference or source
E. coli strain		
DH5α	*F*⁻ *λ*⁻ *ϕ80dLac*ZΔM15 Δ(*lacZYA*-*argF*)U169 *recA*1 *endA*1 *hsdR*17 (rK⁻ mK⁺) *supE*44 *thi-1gyrA relA*1	Takara bio, Kusatsu, Japan
S17-1	*thi pro hsdR*⁻ *hsdM*⁺ *recA* [chr::RP4-2-Tc::Mu-Km::Tn7]	Schafer et al. (1994) [29]
P. cannabina pv. *alisalensis* (*Pcal*)		
Isolate KB211	Wild type, Rif^{r}	Nagano vegetable and ornamental crops experiment station
hexR mutant	*Pcal hexR* mutant, Rif^{r}, Km^{r}	Sakata et al. (2019) [20]
hexR-complemented	*Pcal hexR* mutant containing pDSKG-*hexR*, Rif^{r}, Km^{r}, Gn^{r}	This work
P. syringae pv. *tomato* (*Pst*)		
Isolate DC3000	Wild type, Rif^{r}	From Dr. Katagiri
Δ*hexR*	*Pst hexR* mutant, Rif^{r}	This study
Plasmid		
pGEM-T Easy	Cloning vector, Amp^{r}	Promega, Tokyo, japan
pK18*mobsacB*	Small mobilizable vector, Km^{r}, sucrose sensitive (*sacB*)	Schafer et al. (1994) [29]
pDSKG-*hexR*	The vector containing constitutive *psbA* promoter and *hexR* gene inserted into pDSKG, Gn^{r}	This work

Amp^{r} ampicillin resistance, Gn^{r} gentamicin resistance, Km^{r} kanamycin resistance, Rif^{r} rifampicin resistance

Table S2. Primer sets used in this study

Gene	Primer name	Primer usage	Primer sequence	Reference
Pst hexR	PSPTO_1299-1	Deletion mutagenesis of *Pst hexR*	GGCCAGTGCCTTGAGCACTT	This study
Pst hexR	PSPTO_1299-2	Deletion mutagenesis of *Pst hexR*	TGCAACGCTTCTTCCGATCC	This study
Pst hexR	PSPTO_1299-3	Deletion mutagenesis of *Pst hexR*	CGGGATCCAGGTTTCTTACGCGGTCCAT	This study
Pst hexR	PSPTO_1299-4	Deletion mutagenesis of *Pst hexR*	CGGGATCCGATCGAGGATCAGGGCTGA	This study
Pcal hrpL	hrpL-FW	Expression analysis on *Pcal*	CATGCCAGTAAACCGCAAAC	Sakata et al. (2021) [36]
Pcal hrpL	hrpL-RV	Expression analysis on *Pcal*	GTCTTCCCAGCTTTCCTGATAA	Sakata et al. (2021) [36]
Pcal avrPto	avrPto-FW	Expression analysis on *Pcal*	CATCGATACCTGACCAACGATAC	This study
Pcal avrPto	avrPto-RV	Expression analysis on *Pcal*	CTCAGTCAGCTTGGTGCTAAT	This study
Pcal hopM1	hopM1-FW	Expression analysis on *Pcal*	GGTGCCGATGAAGGCTATT	This study
Pcal hopM1	hopM1-RV	Expression analysis on *Pcal*	TTTCGACGGACGCTTTGT	This study
Pcal avrE1	avrE1-FW	Expression analysis on *Pcal*	GACAATCAGGGCAGGCTTTA	This study
Pcal avrE1	avrE1-RV	Expression analysis on *Pcal*	TGACCTGCCAAGGTGTAATG	This study
Pcal corR	corR-FW	Expression analysis on *Pcal*	GGATCGAACGCTGGCAGATA	Sakata et al. (2021) [36]
Pcal corR	corR-RV	Expression analysis on *Pcal*	GTCCTGCTCATGAGTCGCTT	Sakata et al. (2021) [36]
Pcal cfl	cfl-FW	Expression analysis on *Pcal*	GAACTGGTGGCGTTGTACTAT	Sakata et al. (2021) [36]
Pcal cfl	cfl-RV	Expression analysis on *Pcal*	GTGGAGCAGATGCTCAATTTC	Sakata et al. (2021) [36]
Pcal cmaA	cmaA-FW	Expression analysis on *Pcal*	AAAGCCTACCGCCGATTT	Sakata et al. (2021) [36]
Pcal cmaA	cmaA-RV	Expression analysis on *Pcal*	CGTCTGGAGCTGTTGATAAGT	Sakata et al. (2021) [36]
Pcal oprF	oprF-FW	Expression analysis on *Pcal*	GGCTTGGCCATTGGTACTAT	Sakata et al. (2021) [36]
Pcal oprF	oprF-RV	Expression analysis on *Pcal*	GCGCTGTCGTAATACTCTTTCT	Sakata et al. (2021) [36]
Pcal recA	recA-FW	Expression analysis on *Pcal*	TCTCTACGGCAAGGGTATCT	Sakata et al. (2021) [36]
Pcal recA	recA-RV	Expression analysis on *Pcal*	GCTTTACCCTGACCGATCTT	Sakata et al. (2021) [36]

BamHI digestion sites are underlined.
